# Case series of drug-induced open bite: Extrapyramidal symptoms related to psychotropic medications

**DOI:** 10.3389/fpsyt.2023.1137917

**Published:** 2023-03-28

**Authors:** Motoko Watanabe, Trang Thi Huyen Tu, Chihiro Takao, Chizuko Maeda, Gayatri Krishnakumar Nayanar, Risa Tominaga, Yasuyuki Kimura, Miho Takenoshita, Tatsuya Yoshikawa, Koji Sumi, Satoko Sumi, Haruhiko Motomura, Takahiko Nagamine, Akira Toyofuku

**Affiliations:** ^1^Department of Psychosomatic Dentistry, Graduate School of Medical and Dental Sciences, Tokyo Medical and Dental University, Tokyo, Japan; ^2^University of Medicine and Pharmacy at Ho Chi Minh City, Ho Chi Minh City, Vietnam; ^3^Sumi Orthodontic Clinic, Saga, Japan; ^4^Section of Pediatric Dentistry, Department of Oral Growth and Development, Fukuoka Dental College, Fukuoka, Japan; ^5^Department of Psychiatric Internal Medicine, Sunlight Brain Research Center, Yamaguchi, Japan

**Keywords:** drug-induced open bite, extrapyramidal symptoms, psychotropic medications, antipsychotic medications, antidepressants, schizophrenia, depression, malocclusion

## Abstract

**Introduction:**

Drug-induced open bite is one of the extrapyramidal symptoms with abnormal tonus of muscles and is rarely recognized in dentistry. This is a retrospective case study to investigate clinical characteristics including detailed complaints in patients with drug-induced open bite.

**Methods:**

Of the outpatients who first visited the psychosomatic dental clinic at the Tokyo Medical and Dental University Hospital between September 2013 and September 2022, the patients diagnosed with drug-induced open bite were involved in this study. The clinical characteristics including sex, age, detailed complaints, duration of illness, abnormal findings, psychotropic medications, and other medications that were taken at the first examination, psychiatric comorbidities, the duration of psychiatric diseases, and other medical histories were collected retrospectively by reviewing their medical chart.

**Results:**

Drug-induced open bite was found in 11 patients [women: 7, men: 4, median of age: 49 (36.5, 53) years old]. Difficulty in eating especially chewing was the major complaint (9/11, 81.6%) with the duration of illness as 48.0 (16.5, 66) months. Various degrees of open bite were observed. While some showed no occlusal contact on frontal teeth, some showed occlusal contact only on the second molars; moreover, the jaw showed a horizontal slide in a few patients. Three cases could be followed up for prognosis; while in one case the drug-induced open bite improved with 6 months of follow-up, two cases did not improve, and one showed extrusion of molars. All of them had psychiatric comorbidities with the most common diagnosis being schizophrenia (*n* = 5) and depression (*n* = 5) followed by insomnia (*n* = 1) and autism spectrum disorder (*n* = 1) including duplicated diagnosis. Nine patients (81.6%) had been undergoing treatment with antipsychotics of which three patients were also taking antidepressants.

**Discussion:**

Although a drug-induced open bite is a rare symptom, prudent medical interviews about symptoms, psychiatric comorbidities, and psychotropic medication history besides oral assessment are necessary to provide a precise diagnosis and appropriate management in collaboration between dentists and psychiatrists.

## 1. Introduction

Drug-induced open bite is defined with clinical findings of open bite malocclusion with tonus of masticatory muscles as dystonia (1) without genetic factors in family histories. Dystonia is one of the extrapyramidal symptoms (EPS) and a disorder of abnormal posture and movement due to persistent muscle contraction that is characterized by simultaneous contraction of the dominant and antagonist muscles (co-contraction). Drug-induced open bite is a rare symptom in dentistry but decreases patients' quality of life since it directly affects the masticatory function and has a big impact on their daily diet.

Since drug-induced open bite is a rare symptom, there is a limit to discussing the pathology. However, it has been considered to be associated with the use of antipsychotics (2–4); moreover, antidepressants have also been reported as a cause of EPS (5). In the study of EPS in oral regions at a Japanese dental clinic in a psychiatric hospital, malocclusion was found in 17.6% and was observed with an open bite in some patients (1). The clinical characteristics, including detailed complaints and clinical abnormal findings, in addition to medication and psychiatric history, are important to define drug-induced open bite.

This case study aimed to investigate clinical characteristics including detailed complaints in the patients with drug-induced open bite considering their management through a literature review.

## 2. Methods

Of the out-patients who first visited the psychosomatic dental clinic at the Tokyo Medical and Dental University Hospital between September 2013 and September 2022, the patients with drug-induced open bite were involved in this study. For the definition of drug-induced open bite, the inclusion criteria are open bite malocclusion which shows the space between upper and lower teeth when molars are engaged and the presence of medication history of antipsychotics or antidepressants; and exclusion criteria are open bite induced by other factors including oral habits such as tongue protrusion, thumb sucking, mouth breathing, and so on, and not by medications.

The clinical characteristics including demographic data (sex and age), detailed complaints, duration of illness, abnormal clinical findings, the psychotropic medication that was taken at the first examination, psychiatric comorbidities, and the duration of psychiatric diseases were collected retrospectively by reviewing the medical chart. The diagnoses of comorbid psychiatric disorders were according to referral letters from patients' psychiatrists. All data were described as median (first quantile, third quantile).

All the patients provided written informed consent. This study was approved by the Ethical Committee of the Tokyo Medical and Dental University Hospital (approval number: D2013-005).

## 3. Results

Drug-induced open bite was found in 11 patients [women: 7, men: 4, a median of age: 49 (36.5, 53) years old] out of outpatients who first visited our clinic between September 2013 and September 2022 (Table 1). The median duration of illness was 48.0 (16.5, 66) months. Only three cases could be followed. Difficulty in eating, especially chewing, was the main complaint (9/11, 81.6%), but all 11 patients did not lose their body weight or have any gastrointestinal problems. While two patients complained of occlusal discomfort, the other two patients showed muscle tonus including bruxism and clenching. No patient showed any dystonic symptoms on other body parts.

**Table 1:** Cases with drug-induced open bite.

**Case No.**	**Sex**	**Age**	**Complaints**	**Duration of illness (months)**	**Abnormal findings**	**Psychotropic medication taking at the first visitation**	**Other medication**	**Psychiatric comorbidities**	**Duration of psychiatric comorbidities (years)**	**Other medical histories**
1	M	36	Unstable occlusion Cannot find right occlusal position Cannot bite or chew with frontal teeth	84	Slight open bite (No occlusal ontact from right canine to left canine) Dysfunction of mastication	**Brexpiprazole 1 mg 1T**	None	Schizophrenia	12	None
2	F	46	Difficulty of chewing with enough bite force Jaw has been pulled back Toothache	240	Slight open bite (No occlusal contact from right canine to left canine) Mandibular shifted to left Dysfunction of mastication	**Blonanserin 2 mg 1T** Bromazepam 2 mg 3T Flunitrazepam 1 mg 1T	Biperiden 1 mg 1T Chinese harbs: KANPO	Schizophrenia	23	Hyperlipidemia
3	F	54	Difficulty of chewing with molars Mucus membranes are coming off	9	Slight open bite (No occlusal contact from 2nd premolar to lateral incisor on either side) Dysfunction of mastication	Paroxetine 25 mg 1T, 12.5 mg 1T Mirtazapine 15 mg 1T Flunitrazepam 1 mg 1T Zolpidem 10 mg 1T	Linzess 0.25 mg 1 T Sodium Picosulfate Hydrate 2.5 mg 3T Letrozole 2.5 mg 1T	Depression	0.8	Breast cancer
4	M	52	Bite is sliding especially on left molars Mouth have been deformed Cannot bite tightly	37	Slight open bite (No occlusal contact from 1st right premolar to left 1st premolar including frontal teeth)	**Perospirone 4 mg 1T** Flunitrazepam 1 mg Eszopiclone 3 mg 1T	Biperiden 1 mg 2T	Obsessive-compulsive disorder Schizophrenia	15	Hyperlipidemia Renal cyst
5	F	50	Difficulty of chewing Can bite but cannot grind foods	24	Slight open bite (No occlusal contact from right 1st premolar to left 1st premolar including frontal teeth) Mandibular slightly shift to right Dysfunction of mastication	**Sulpiride 50 mg 1T Qetiapine 25 mg 4T** Zolpidem 10 mg 1T Bromazepam 5 mg 1T	Bromocriptine 2.5 mg 2T Clonazepam 0.5mg 1T Magnesium oxide 330 mg 2T	Depression	0.5	Hyperlipidemia
6	M	57	Cannot get contact between upper and lower teeth Difficulty of chewing	48	Open bite (No occlusal contact from right 2nd premolar to left 2nd premolar including frontal teeth) Mandibular shifte to right Dysfunction of mastication	**Aripiprazole 12 mg 2T, 6 mg 1T** Mirtazapine 15 mg 2T Flunitrazepam 2 mg 1T Zolpidem 10 mg 1T	Biperiden 1 mg 2T Pioglitazone 15 mg 2T Sitagliptin Phosphate Hydrate 50 mg 1T Amlodipine 2.5 mg 1T Pravastatine Sodium 5 mg 1T	Depression	29	Hyperlipidemia Diabetes millitus
7	F	59	Cannot bite off foods with frontal teeth	72	Open bite (No occlusal contact from right 1st premolar to left 1st premolar including frontal teeth) Dysfunction of mastication	**Levomepromazine 5 mg 1T Vegetamin A**^®^ **2T** Fluvoxamine 75 mg 1T, 25 mg 2T Mianserin 30 mg 1T Etizolam 0.5 mg 1T Pentobarbital 50mg 1T Flurazepam 15 mg 2C	Teprenone 50 mg 2C Befotiamine 25 mg 3C Hydroxyzine Hydrochloride 25 mg 1C	Depression	15	Irritable bowel syndrome Hyperlipidemia
8	F	49	Involuntary movement of jaw which is reduced during smoking Muscle tonus around mandibule	60	Open bite (No occlusal contact from right 1st molar to left 1st premolar including frontal teeth) Involuntary movement of mandibular and tongue	**Levomepromazine 25 mg 1T Lurasidone hydrochloride 40 mg 1T** Etizolam 0.5 mg 1T Flunitrazepam 1 mg 2T Lemborexant 5 mg 1T	Biperiden 1 mg 2T	Schizophrenia Insomnia	6	None
9	M	37	Cannot bite off with frontal teeth Difficulty of chewing especially noodles sounds of temporomandibular joints	12	Open bite (Occlusal contact on left 1st molar and right 2nd molars) Click of both sides of temporomandibular joints	**Quetiapine 25 mg 1T Lisperidone 2 mg 1T** Brotizolam 0.25 mg 1T Flunitrazepam 1 mg 1T Lorazepam 1 mg 1 T	Rosuvastatin 5 mg 1T	Schizophrenia	19	Hyperlipidemia
10	F	18	Jaw became protrusive Difficulty of chewing meat	55	Open bite (Occlusal contact only on both sides of second molar) Mandibular shifted to right Dysfunction of mastication Mandibular protrusion	**Quetiapine fumarate 100 mg 3T Olanzapine 5 mg 1T Risperidone 1 mg 1T** Escitalopram 10 mg 1T Trazodone 50 mg 2T Venlafaxine 75 mg 3C Nitrazepam 10 mg 1T, 5 mg 1T	Biperiden 1 mg 2T Lubiprostone 12 μg 2C Sennoside 12 mg 3T	Autism spectrum disorder	8	None
11	F	19	Opened frontal teeth in few months Jaw has been pulled back Difficulty of eating and moving tongue Clenching and teeth chattering	15	Open bite (Occlusal contact only on both side of second molar) Dysfunction of mastication Hypermiotonia of masticatory muscle	None (Use to take Fluvoxamine 25 mg 3T)	Clonazepam 0.5 mg 1T	Persistent depressive disorder	8	Allergic dermatiti

Medications with bold indicate antipsychotics. Medications with underline indicate serotonin reuptake inhibitors (SRI) or selective SRI. Vegetamine^®^ (Shionogi & CO., LTD. Osaka, Japan) tablet contains phenobarbital (40 mg), chlorpromazine hydrochloride (25 mg), and promethazine hydrochloride (12.5 mg).

**Figure 1:**
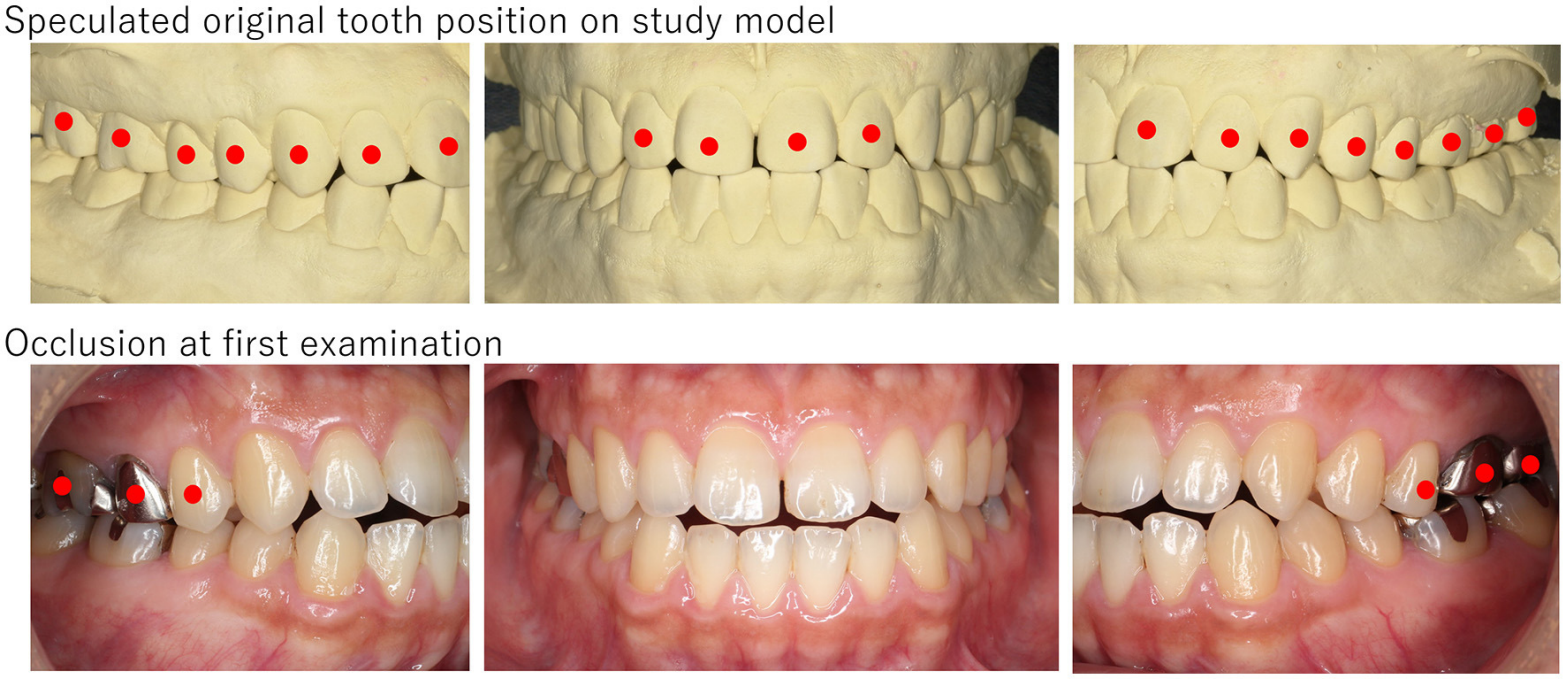
The picture of a patient (Case No. 1). Slight open bite was observed with no occlusal contact from the right canine to the left one. Since facets were observed on both sides of the canine and incisal edges and his study model showed tight occlusion, his mandibular had potentially shifted anteroinferior. Red circles indicate teeth that have occlusal contact.

**Figure 2:**
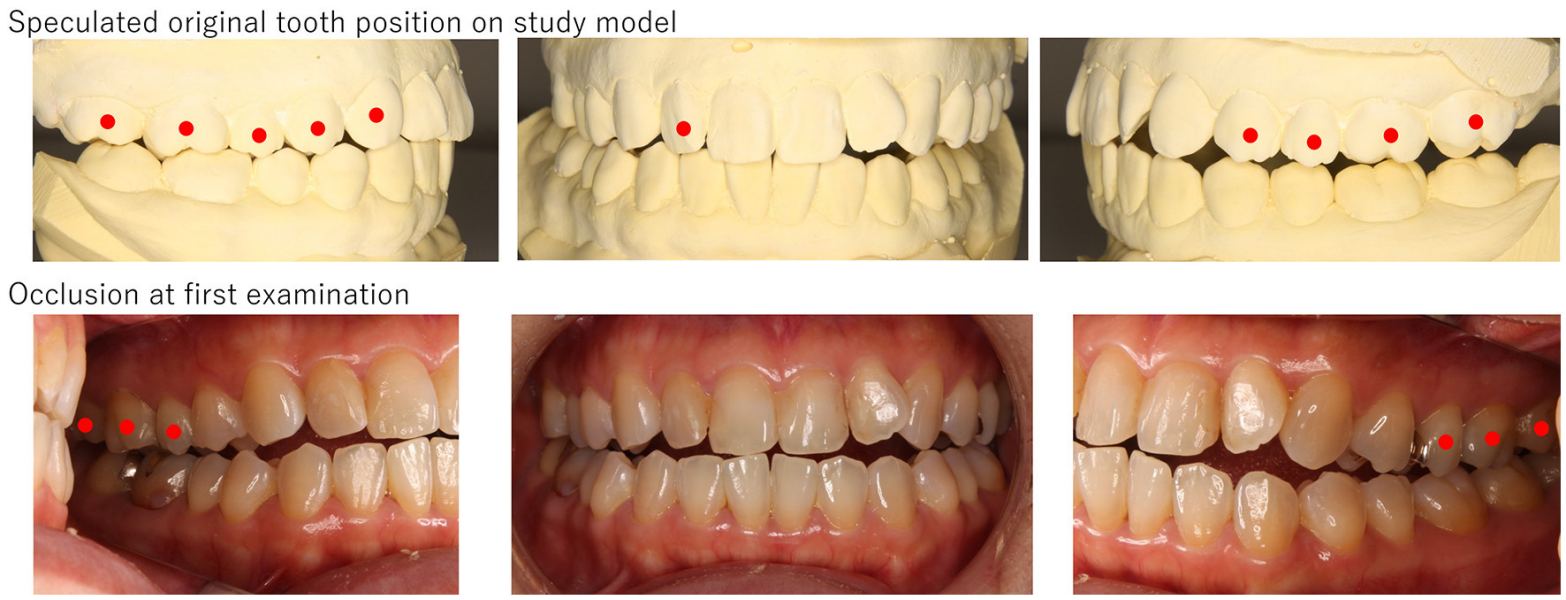
The picture of a patient (Case No. 5). Slight open bite was observed with no occlusal contact from the right first premolar to the left first premolar, and the mandibular slightly shifted to the right. The presence of facets and reconstruction on the study model revealed her mandibular shifted to anteroinferior, and consequently, open bite was presented. Red circles indicate teeth which have occlusal contact.

**Figure 3:**
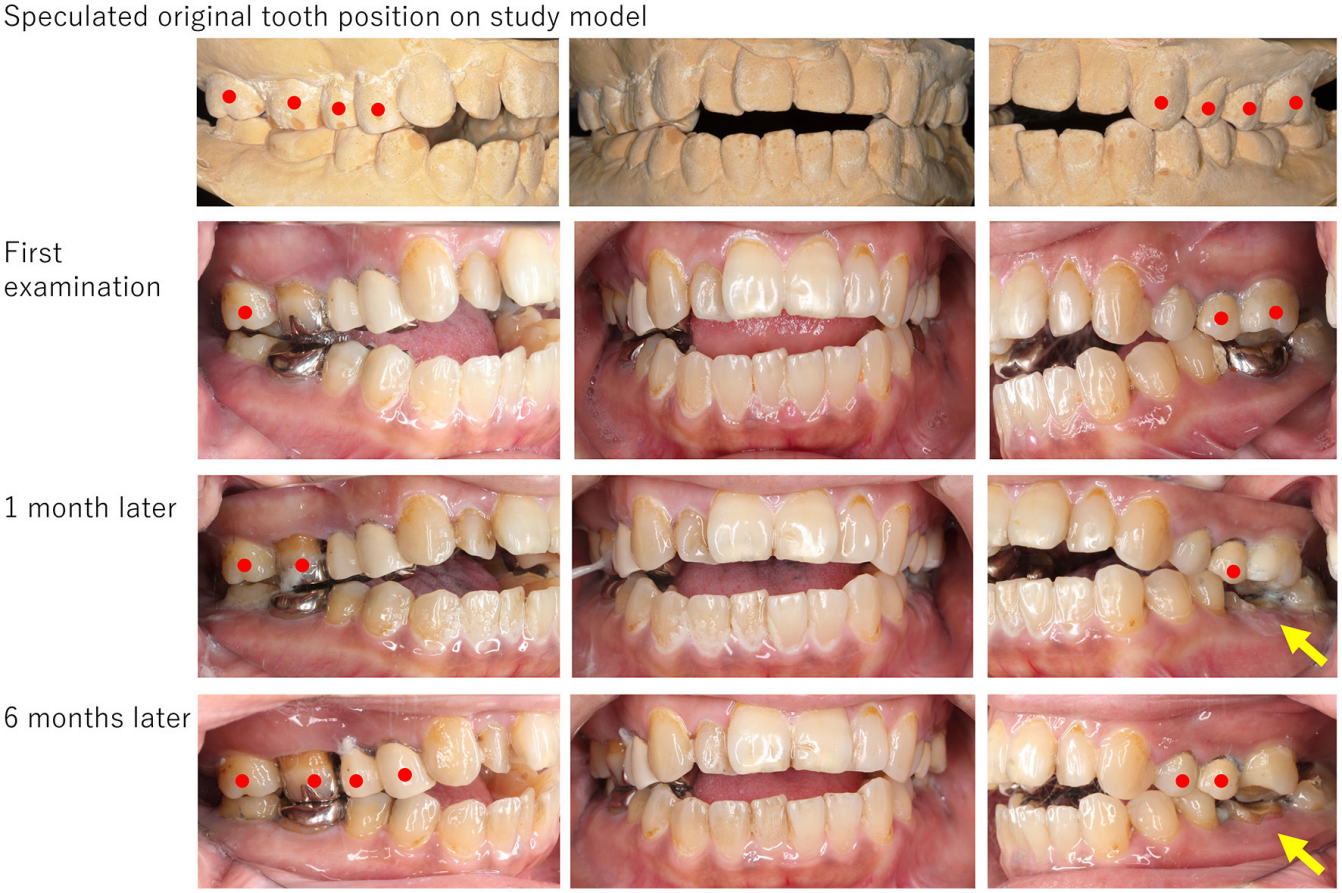
The picture of a patient (Case No. 8). Open bite was obvious, but occlusal contact was observed on the right second molar, left second premolar, and left molars. Frontal teeth showed open bite even on the study model; however, the left canine and both sides of the premolars and molars engaged tightly. After 6 months of follow-up, drug-induced open bite improved gradually close to the occlusion observed in the study model. Although the dental crown on the lower left first molar had been removed for the root canal treatment (yellow arrows), any other dental procedures including occlusal adjustment had not been presented. Red circles indicate teeth that have occlusal contact.

**Figure 4:**
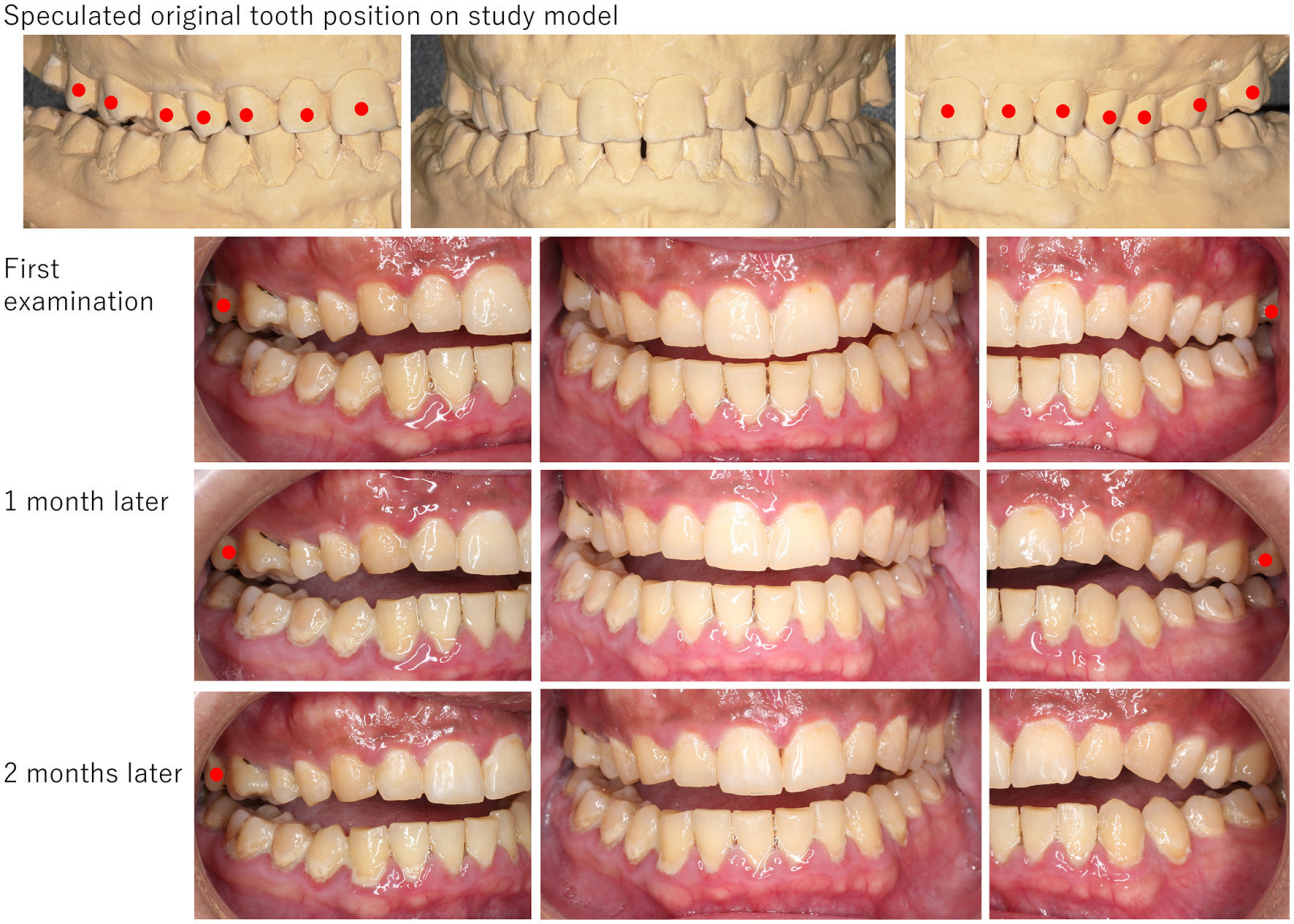
The picture of a patient (Case No. 10). Open bite was obvious, and occlusal contact was observed only on the second molars. Tight occlusal contact was found on the left premolars to right molars on his study model, but partially on the right molars. Drug-induced open bite had never changed during 2 months of follow-up. Red circles indicate teeth that have occlusal contact.

**Figure 5:**
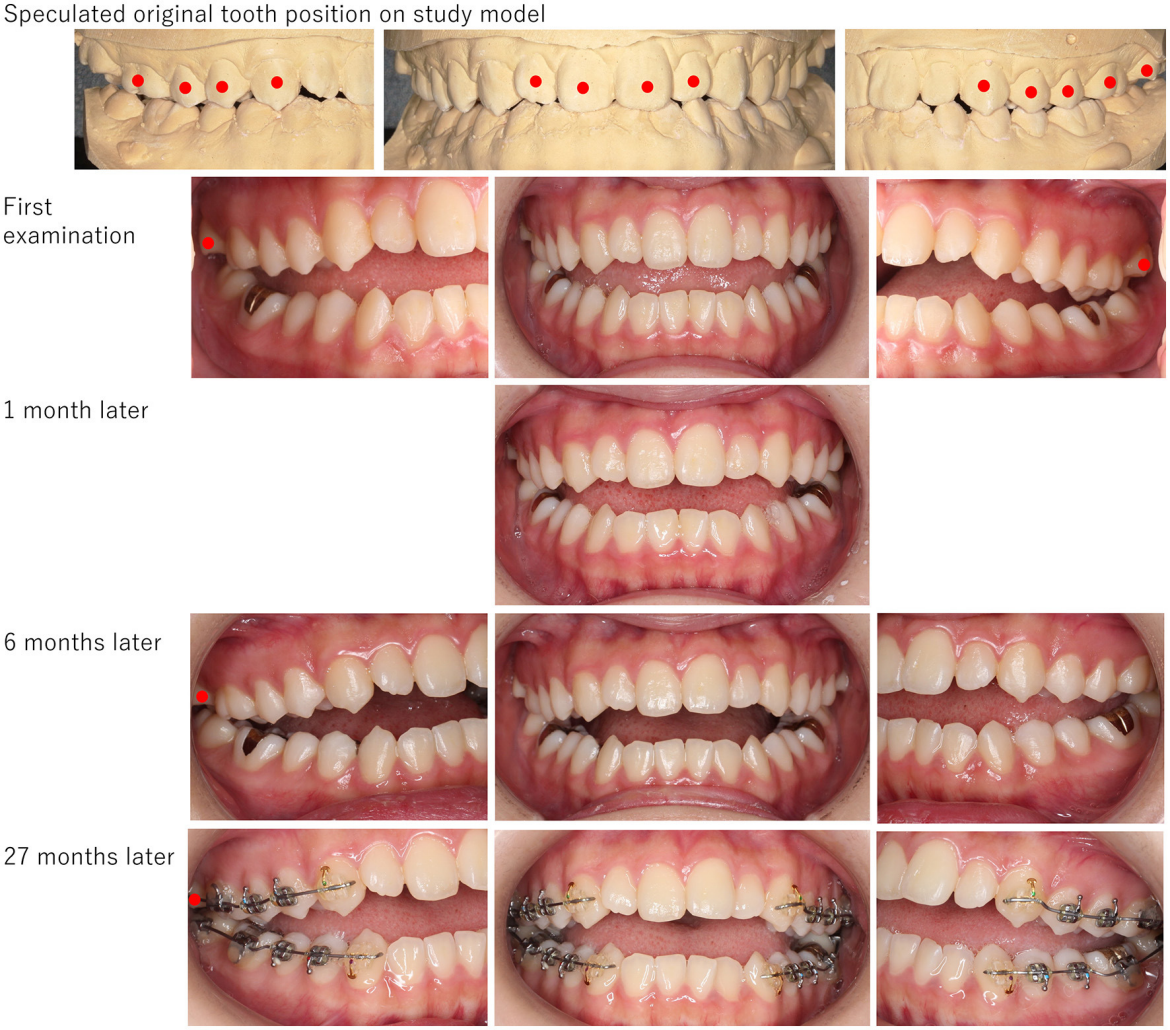
The picture of a patient (Case No. 11). Open bite was obvious, and occlusal contact was observed only partially on the second molars. Tight occlusal contact was found on her study model. Drug-induced open bite never improved even after stopping psychotropic medications. The orthodontic treatment for the intrusion of the second molars started 27 months later. Red circles indicate teeth that have occlusal contact.

The open bite in patients was of various degrees. In the patients who showed slight open bite (Case No. 1–Case No. 5), some could hardly recognize their symptoms exactly. They complained of difficulty in chewing or occlusal discomfort alone. The patient in Case No. 3 expressed that “the mouth has been deformed”. As for abnormal clinical findings, Case No.1 showed no occlusal contact from the right canine to the left one; however, his study model which indicated a speculated original tooth position, showed tight occlusion including frontal teeth (Figure 1). Similarly, Case No. 5 showed a slight open bite with no occlusal contact from right first premolar to left first premolar (Figure 2). However, her study model showed tight occlusal contact on her right molars and partially on the left side, and facets were found on upper and lower right canine and upper right second incisor. These findings possibly suggest that her mandibular could have shifted anteroinferior and consequently led to an open bite. The cases from Case No. 6 to Case No. 8 showed obvious open bite but had occlusal contact on premolars. For Case No.8, her frontal teeth showed no contact even on the study model but the left canine and both sides of her molars engaged tightly (Figure 3). Since her psychiatric condition was unstable and psychotropic medications were also unfixed, we kept her under timely follow-up along with training of the mastication muscles. One month later, the right first premolar contact was established; moreover, spaces between the canines had also started getting narrower. Although the dental crown on the lower left first molar had been removed for the root canal treatment, no other dental procedures including occlusal adjustment had been presented. Her occlusion got close to the one shown on the study model 6 months later. Drug-induced open bite without occlusal contact on molars was observed in cases No. 9–No. 11, and they recognized malocclusion with complaints such as “occlusion gradually changed over a year”, “jaw has protruded”, and “opened frontal teeth in few months”. All of them were recommended to have orthodontic treatment by general dentists and they required it strongly owing to their chewing difficulty. After a few months of follow-up, their occlusions never improved (Figures 4, 5). Case No. 11 was referred to neurologists since the patient and her parents wanted to have treatment with botulinum injection. Twenty-seven months after her first examination, she revisited our department and told us that botulinum injection was not prescribed for some reason. Moreover, although the tonus of masticatory muscles, bruxism, and clenching improved, both sides of her second molars had been elongated (Figure 5). No improvement was observed, instead extraction of her wisdom teeth, and orthodontic treatment for the intrusion of the second molars were started in consultation with her psychiatrist at that time.

All the patients had psychiatric comorbidities with the most common diagnosis being schizophrenia (*n* = 5) and depression (*n* = 5) followed by insomnia (*n* = 1) and autism spectrum disorder (*n* = 1) including duplicated diagnosis. The median duration of psychiatric disorders was 12 (6, 7) years. Nine patients (81.8%) had been undergoing treatment with antipsychotics of which 3 patients were taking antidepressants. As for the typical antipsychotics in this study, levomepromazine, vegetamine A^®^ [Shionogi & CO., LTD. Osaka, Japan, which contains phenobarbital (40 mg), chlorpromazine hydrochloride (25mg), promethazine hydrochloride (12.5mg)], and sulpiride were found to be used by three patients. Dopamine partial agonist (DPA) (aripiprazole and brexiprazole) was being taken by two patients, serotonin and dopamine antagonist (SDA) (perospirone, blonanserin, lurasidone, and risperidone) was being taken by 5 patients, and multi-acting receptor targeted antipsychotics (MARTA) (quetiapine and olanzapine) were being taken by three patients. Four patients were under more than two types of antipsychotics. Antidepressants, noradrenergic, and specific serotoninergic antidepressants (NaSSA) were used by two patients, serotonin selective reuptake inhibitors (SSRI) and serotonin reuptake inhibitors (SRI) by four patients including a patient who had been taking it for years; moreover, two patients were taking only antidepressants.

## 4. Discussion

Eleven patients with drug-induced open bite were investigated in this study. The major complaints were difficulty in eating, especially chewing, and occlusal discomfort. While only a few patients were under antidepressants and anxiolytics, most of them were under antipsychotics, mostly diagnosed with schizophrenia or depression for years.

Drug-induced open bite is considered one of the dystonia caused by EPS. The most accepted mechanism is related to dopamine receptors in EPS, especially D2 receptors whose decrease causes Parkinson's syndrome and increase is associated with schizophrenia. Antipsychotic drugs are D2 receptor blockers and are representative of drugs that put patients at high risk for developing EPS (6) and drug-induced open bite (1, 7). Generally, a blockade of about 80% or more of D2 receptors produces EPS. Because of the potent D2 blockade of typical antipsychotics, the risk of EPS has been considered higher than with atypical antipsychotics. However, the dose used is rather influential. In recent years, the risk of developing drug-induced parkinsonism with typical and atypical antipsychotics has not changed significantly at doses that result in approximately equal D2 receptor occupancy. Clozapine, which occupies only approximately 60% of D2 receptors at clinical doses, is an atypical antipsychotic that is unlikely to cause EPS based on receptor occupancy. Aripiprazole is a type of DPA that has less agonist action to D2 receptors than typical antipsychotics; however, it also induces drug-induced open bite (4). Atypical antipsychotics were observed more than typical ones in this study. Given the expanding use of atypical antipsychotics, drug-induced open bite also should be considered besides other EPS. As for the duration of psychiatric diseases, many of the patients were under medication for over 10 years although the data on the exact duration for each medication is lacking. Whether it was an acute drug-induced open bite or a tardive one may affect its prognosis. Further studies with more detailed medication data of more samples are needed.

Among atypical antipsychotics, atypical antipsychotics with serotonin 2A (5HT2A) receptor blockade have been considered less likely to cause EPS because 5HT2A blockade releases dopamine (5). However, dopamine release by serotonin blockade and atypical antipsychotics at higher doses strengthen dopamine D2 receptors and cause EPS. Furthermore, 5HT2A receptor blockade, when considered at the cellular level, decreases intracellular signaling in dopamine neurons, so it cannot be simply said that 5HT2A receptor blockade makes a patient less likely to develop EPS. The substantia nigra striatum contains a number of serotonergic systems. In particular, 5HT2A receptors are known to inhibit dopamine release in the substantia nigra and striatum. Thus, there is a risk of drug-induced parkinsonism by stimulating 5HT2A receptors. Antidepressants such as tricyclic antidepressants and serotonin reuptake inhibitors (SSRIs) increase serotonin levels in the brain by inhibiting serotonin reuptake, thus risking the induction of drug-induced parkinsonism. SSRI showed a significantly high prevalence of EPS compared to other groups of antidepressants (5), and it had often been found as a cause of drug-induced bruxism (5, 8). In the present cases, paroxetine, fluvoxamine, escitalopram, and trazodone were found, and a patient with fluvoxamine complained of clenching and bruxism. Moreover, combination therapies with antipsychotics and antidepressants were observed in some presented cases. There are potential problems with a combination of antipsychotic and antidepressant therapy. A majority of these medications are primarily metabolized by cytochrome P450 2D6 (CYP2D6) and 3A4 (CYP3A4) in the liver. SRIs also inhibit the activity of CYP2D6 and CYP3A4. As a result, there is a risk of increased blood levels of each, resulting in stronger dopamine blockade (9). Although the mechanisms are not precisely known, direct and indirect inhibition of the dopaminergic system might be involved in a drug-induced open bite.

According to these suggested mechanisms, various antipsychotics and antidepressants interacted in a complicated manner and induced open bite in this study. Since most complaints in this study were related to difficulty in chewing rather than bruxism, more complicated muscles' tonus reflecting co-contractions of opening and jaw-closing muscles (1) might be involved in drug-induced open bite, while bruxism and clenching are involved in the hypertonic of jaw-closing muscles. In this study, patients' mandibles tended to be sifted to anteroinferior and left or right. These clinical phenomena suggested that the external pterygoid muscle which relates to jaw movement to the anterior and side, and masseter muscles which are activated during the closing of the mouth are also involved. The asymmetric tonus of these muscles and the intensity of tonus might be related to the degree of drug-induced open bite, although the detailed mechanisms for drug-induced open bite remain unclear.

Interestingly, while male to female ratio was found to be equal in the age group of 10–30 s, a female predominance was observed over the age of 40 years. Dystonia is often found in young men but increases in women with aging (10). The D2 receptor density and binding potential in the striatum reduce with age; moreover, a lower affinity of the D2 receptors in women with a greater decline in aging than in men has been reported (11). In the terms of morphological sex differences, the masticatory function is stronger in young men than women resulting in stronger muscle force in males (12). Drug-induced open bite might have different frequencies related to sex and age reflecting various factors including the different degrees of dopaminergic responses to psychotropic medications.

For the treatment of drug-induced open bite, since the withdrawal of the medication may exacerbate comorbid psychiatric disorders, the pharmacological treatment is very sensitive. For acute dystonia, anticholinergic medication is effective, but the treatment strategy has not been established for tardive dystonia except clonazepam (2), botulinum injection (13), and selective vesicular monoamine transporter 2 (VMAT2) inhibitors (13). Besides pharmacological management, prudent consideration for dental procedures is also necessary. When patients showed slight drug-induced open bite without exact recognition, the definition of drug-induced open bite would be difficult, and some might be misdiagnosed only as dental problems (14). Prudent medical interviews including medications and psychiatric histories are critical; moreover, the existence of facets and reconstructing occlusions on study models would be useful evidence to confirm their occlusion in the past. Most importantly, irreversible procedures including occlusal adjustment, prosthodontic reconstruction, or orthodontic treatment should be avoided. Even if occlusal discomfort seems to be improved by dental procedures, it would be just for a limited time. It would continue to change according to changes in the muscles' tonus during the period from when the open bite is found until its improvement. To make matters worse, invasive treatments might exacerbate the psychiatric condition and cause iatrogenic dental problems (15). Furthermore, some patients did not complain about their uncomfortable symptoms in the oral region to their psychiatrists since they thought those were dental problems, and some even did not exactly recognize the occlusal changes. Therefore, dentists would be the first to find the issue. For complaints relating to difficulty in chewing, occlusal discomfort, and bruxism with open bite, dentists should consider psychiatric comorbidities and medication histories to provide appropriate treatments. Follow-up in dentistry with prudent psychiatric treatment including pharmacological management may ameliorate occlusal changes without invasive dental procedures (3, 4) as seen in Case No. 8 in this study.

At the same time, a drug-induced open bite may accompany tooth movement. For example, besides change of occlusal presser through the long-term duration of psychiatric diseases, dyskinesia of the tongue as another EPS might produce malocclusion. Moreover, the onset of schizophrenia is commonly diagnosed in adolescence, the age when the impacted wisdom tooth eruption might also affect tooth movements. In such cases, even after improvement of drug-induced open bite, their occlusion would not recover to what it used to be. As seen in Case No. 11, some patients would require necessary additional dental procedures including orthodontic treatments after the drug-induced open bite has improved while working together with their psychiatrists. Further studies with longer follow-ups are critical, especially for patients considering dental procedures.

There are a few limitations in this study. First, since clinical follow-up was missing, the data for prognosis are lacking. Second, the small sample size is also a limitation of this study. Further investigation with a larger sample size and longer follow-up is necessary.

In conclusion, 11 patients with drug-induced open bite were identified at the oral psychosomatic dental clinic. The complaints of difficulty chewing were mainly found with various degrees of open bite. Although drug-induced open bite is a rare symptom, prudent medical interviews about symptoms, psychiatric comorbidities, and psychotropic medication history, besides oral assessment are necessary to provide a precise diagnosis and appropriate management in collaboration between dentists and psychiatrists.

## Data availability statement

The original contributions presented in the study are included in the article, further inquiries can be directed to the corresponding author.

## Ethics statement

The studies involving human participants were reviewed and approved by the Ethical Committee of Tokyo Medical and Dental University Hospital. The patients provided their written informed consent to participate in this study. Written informed consent was obtained from the individual(s) for the publication of any potentially identifiable images or data included in this article.

## Author contributions

MW participated in data acquisition and analysis and writing manuscript. TT, KS, and SS reviewed and edited the manuscript. CT, CM, GN, RT, YK, MT, and TY participated in collecting data. HM, TN, and AT developed the theory and supervised the research. AT designed the research and administered the project. All authors read and approved the final manuscript.
